# Prime-boost vaccination of mice and rhesus macaques with two novel adenovirus vectored COVID-19 vaccine candidates

**DOI:** 10.1080/22221751.2021.1931466

**Published:** 2021-06-01

**Authors:** Shengxue Luo, Panli Zhang, Bochao Liu, Chan Yang, Chaolan Liang, Qi Wang, Ling Zhang, Xi Tang, Jinfeng Li, Shuiping Hou, Jinfeng Zeng, Yongshui Fu, Jean-Pierre Allain, Tingting Li, Yuming Zhang, Chengyao Li

**Affiliations:** a Department of Pediatrics, Shenzhen Hospital, Southern Medical University, Shenzhen, People’s Republic of China; b Department of Transfusion Medicine, School of Laboratory Medicine and Biotechnology, Southern Medical University, Guangzhou, People’s Republic of China; c Guangzhou Bai Rui Kang (BRK) Biological Science and Technology Limited Company, People’s Republic of China; d School of Pharmaceutical Sciences, Southern Medical University, Guangzhou, People’s Republic of China; e Department of Infection, The First People’s Hospital of Foshan, Foshan, People’s Republic of China; f Shenzhen Key Laboratory of Molecular Epidemiology, Shenzhen Center for Disease Control and Prevention, Shenzhen, People’s Republic of China; g Guangzhou Center for Disease Control and Prevention, Guangzhou, People’s Republic of China; h Shenzhen Blood Center, Shenzhen, People’s Republic of China; i Guangzhou Blood Center, Guangzhou, People’s Republic of China; j Emeritus Professor, University of Cambridge, Cambridge, UK.

**Keywords:** COVID-19 vaccines, simian adenovirus 23 vector, human adenovirus 49 vector, prime-boost vaccination, mice and non-human primates

## Abstract

COVID-19 vaccines are being developed urgently worldwide. Here, we constructed two adenovirus vectored COVID-19 vaccine candidates of Sad23L-nCoV-S and Ad49L-nCoV-S carrying the full-length gene of SARS-CoV-2 spike protein. The immunogenicity of two vaccines was individually evaluated in mice. Specific immune responses were observed by priming in a dose-dependent manner, and stronger responses were obtained by boosting. Furthermore, five rhesus macaques were primed with 5 × 10^9^ PFU Sad23L-nCoV-S, followed by boosting with 5 × 10^9^ PFU Ad49L-nCoV-S at 4-week interval. Both mice and macaques well tolerated the vaccine inoculations without detectable clinical or pathologic changes. In macaques, prime-boost regimen induced high titers of 10^3.16^ anti-S, 10^2.75^ anti-RBD binding antibody and 10^2.38^ pseudovirus neutralizing antibody (pNAb) at 2 months, while pNAb decreased gradually to 10^1.45^ at 7 months post-priming. Robust T-cell response of IFN-γ (712.6 SFCs/10^6^ cells), IL-2 (334 SFCs/10^6^ cells) and intracellular IFN-γ in CD4^+^/CD8^+^ T cell (0.39%/0.55%) to S peptides were detected in vaccinated macaques. It was concluded that prime-boost immunization with Sad23L-nCoV-S and Ad49L-nCoV-S can safely elicit strong immunity in animals in preparation of clinical phase 1/2 trials.

## Introduction

Novel coronavirus disease 2019 (COVID-19) usually presents as severe acute respiratory syndrome triggered by SARS-CoV-2 infection [1, 2], which has become globally pandemic and more than 3 million people died worldwide (https://covid19.who.int/, 18 April 2021) [3]. Currently the most urgent need is to develop safe, effective, easy production, and distribution vaccines that prevent SARS-CoV-2 infection.

SARS-CoV-2 is a single-stranded positive-sense RNA virus, encoding four structural proteins, including spike (S), envelope (E), membrane (M), and nucleocapsid (N) [4, 5]. The S protein is a glycoprotein carrying the cell receptor binding domain (RBD) and is a major immunogenic antigen that may elicit potent neutralizing antibody (NAb) and cellular immunity [4, 5]. Therefore, S protein has been the primary target antigen for developing recombinant vaccines.

According to the World Health Organization (WHO) report for a draft landscape of COVID-19 candidate vaccines (16 April 2021), there are 88 vaccine candidates in clinical evaluation, which are mainly distributed across five types of biotechnological platforms, *i.e.* inactivated virus, DNA, RNA, protein subunit, and non-replicating viral vector vaccines [6]. Among COVID-19 candidate vaccines in emergency use and clinical trials on 16 April 2021, 10 non-replicating adenovirus vectored vaccines progressed to the frontline, of which 4 (CanSino Biological Inc./Beijing Institute of Biotechnology, Gamaleya Research Institute, University of Oxford/AstraZeneca, Janssen Pharmaceutical) have been registered and approved for emerging use, and 1 has been in phase III clinical trial (ReiThera/LEUKOCARE/Univercells), and 5 in phase I clinical trial (Vaxart, ImmunityBio, Altimmune, Bharat Biotech International Limited, Gritstone Oncology), respectively [6]. Recombinant adenovirus vectors originated from various serotype of strains have displayed good safety profiles and induced broad and strong humoral and cellular immune responses, which have been widely used for research and development of vaccines [7]. Based on the published data regarding human adenovirus type 5 (Ad5), chimpanzee adenovirus (ChAdOx1) and human adenovirus type 26 (Ad26) vectorial COVID-19 vaccines [8–14], results showed that a single-shot vaccine prevented SARS-CoV-2 pneumonia in rhesus macaques and hamsters, and elicited significant immune response in the majority of recipients in phase I/II clinical trials. Enhancement of immune response was evidenced by prime-boost regimen [15], such as homologous boosting of ChAdOx1 nCoV-19 in animals and humans [11, 16], heterologous Ad26 and Ad5 vectored COVID-19 vaccines in humans [17], heterologous Ad26/Ad35 or Ad35/Ad5 vectored human immunodeficiency virus (HIV) vaccines in humans [18, 19], and heterologous Ad6 and ChAd3 vectored hepatitis C virus (HCV) vaccines in humans [20].

In this study, two novel simian adenovirus type 23 (SAdV23) and human adenovirus type 49 (HAdV49) derived vectors (Sad23L and Ad49L) were used for developing the COVID-19 vaccines carrying the full-length S gene of SARS-CoV-2, designated as Sad23L-nCoV-S and Ad49L-nCoV-S vaccines, respectively. These two adenovirus vectors COVID-19 vaccines presented high infectious titers and low frequencies of pre-existing immunity in humans. Immunogenicity was extensively evaluated in mice and rhesus macaques by prime-boost vaccinations with these two novel heterologous adenovirus vectored vaccines.

## Materials and methods

### Rhesus macaques and ethics statement

Eight healthy outbred male rhesus macaques (*Macaca mulatta*) aged 11–14 years were randomly allocated to this study (see Table S1). Experimentation and sample collection were ethically approved and carried out by the Huazheng Laboratory Animal Breeding Centre, Guangzhou, China. All animal care and experimental procedures (NFYYLASOP-037) were in accordance with national and institutional policies for animal health and wellbeing.

The welfare issues (housing, feeding, environmental enrichment, etc.) were in accordance with the recommendations of the Weatherall report (https://acmedsci.ac.uk/more/news/the-use-of-non-human-primates-in-research). Animals were individually housed in spacious cages and were provided with commercial food pellets supplemented with appropriate treats. Drinking water was provided ad libitum from an automatic watering system. Animals were monitored daily for health and discomfort. Blood samples were obtained using sterilized needle and syringe from the venous vessels of animal legs.

### Cells and mice

HEK-293A, HEK-293T, and HEK293T-hACE2 cells (Sino Biological) were maintained in complete Dulbecco's modified Eagle's medium (DMEM, Gibco) supplemented with 10% fetal bovine serum (FBS, Gbico) and incubated at 37°C in 5% CO_2_.

Female C57BL/6 and BALB/c mice were obtained from the Animal Experimental Centre of Southern Medical University, Guangdong, China. All experiments were conducted in compliance with the guidelines for the care and use of laboratory animals and approved by the Southern Medical University (SMU) Animal Care and Use Committee at Nanfang hospital, SMU, Guangzhou, China (permit numbers: SYXK [Yue] 2010-0056).

### COVID-19 patients’ serum samples

A total of 48 convalescent serum samples were provided by The First People's Hospital of Foshan, Shenzhen or Guangzhou Center for Disease Control and Prevention (CDC), China. The samples were collected from 25 asymptomatic, 14 mild and 9 severe COVID-19 infected subjects. All serum samples were inactivated for 40 min by heating at 56°C in the water bath. All patients signed an informed consent for blood sample collection and further laboratory examination. This study was approved by the Medical Ethics Committee of Southern Medical University, The First People's Hospital of Foshan, Shenzhen and Guangzhou Centers for Disease Control and Prevention, and followed the ethical guidelines of the 1975 Declaration of Helsinki.

### Construction of two novel adenovirus vectored COVID-19 vaccine strains

According to the description of Sad23L vector (SAdV23, GenBank: AY530877.1) [21, 22], the replication defective adenoviral vector Ad49L was constructed by deleting the E1 and E3 regions of the full-length human adenovirus serotype 49 genome (HAdV49, GenBank: DQ393829.1). The E4 region open reading frame 6 (orf6) was replaced by the corresponding element of human adenovirus type 5 (Ad5) in order to improve virus propagating efficiency. The full-length S protein gene of SARS-CoV-2 (GenBank: MN908947.3) was optimized and synthesized (Beijing Genomics Institute, China) and was cloned into adenoviral vectors Sad23L and Ad49L, respectively. The recombinant adenoviral constructs Sad23L-nCoV-S and Ad49L-nCoV-S were rescued, and the novel adenovirus vectored COVID-19 vaccine strains were propagated from HEK-293A packaging cells. The vaccine strains were serially passaged for stability by 12 generations when the full cytopathic effect appeared. Virus purification was performed by cesium chloride density gradient centrifugation as previously described [22].

### Histopathological examination

Mice tissues were stained with hematoxylin and eosin (H&E) and examined microscopically for histopathological changes by Guangzhou Huayin Medical Science Company Limited (Guangzhou, China).

### Western blotting

HEK-293A cells were infected with Sad23L-nCoV-S and Ad49L-nCoV-S strains, respectively, and Sad23L-GFP and Ad49L-GFP vectorial viruses were used as mock control. The expression of SARS-CoV-2 S protein was analysed by Western blotting with rabbit polyclonal antibody to SARS-CoV-2 RBD (Sino Biological, 40592-T62, China). Glyceraldehyde-3-phosphate dehydrogenase (GAPDH) was included as a loading control. The membranes were washed five times and developed by Supersignal West Pico Plus chemiluminescent substrate (Thermo Scientific, USA).

### Immunofluorescence staining

Cells infected with vaccine strains or vector control viruses were fixed in cell culture plates, while tissues were collected from vaccinated and control mice. Cell layers or tissue frozen sections were incubated with human monoclonal antibody to SARS-CoV-2 RBD (OkayBio, K79d9, China), and then washed with PBST. Anti-human IgG-Alexa Fluor 594 antibody (Thermo Scientific, USA) in 1% BSA-PBST was added to the cells for 30 min at 37°C. DAPI was added to stain cell nuclei.

### Adenovirus neutralizing antibody (AdNAb) assay

Human plasma samples were collected from 600 healthy blood donors at 6 blood centres (100 per centre) across China, including Shenzhen (south), Guangzhou (south), Yichang (central), Harbin (northeast), Chengdu (southwest), and Xian (west) blood centres. Plasmas were tested on HEK-293A cells for neutralizing Ad5-GFP, Sad23L-GFP, or Ad49L-GFP vectorial viruses by green fluorescent activity assay as previously described [21, 23]. AdNAb titers were defined as the maximum serum dilution that neutralized 50% of Green activity.

### Animal immunization

Female C57BL/6 mice (5–6 weeks, *n* = 5/group) were individually inoculated intramuscularly (i.m.) with a dose of 10^7^, 10^8^, and 10^9^ PFU Sad23L-nCoV-S or Ad49L-nCoV-S vaccine, respectively. A dose of 10^9^ PFU Sad23L-GFP or Ad49L-GFP vectorial virus and an equivalent volume of PBS were used as sham control.

Female C57BL/6 and BALB/c mice (5–6 weeks, *n* = 5/group) were prime inoculated intramuscularly with a dose of 10^9^ PFU Sad23L-nCoV-S vaccine, and then at 4-week interval were boosted with a dose of 10^9^ PFU Ad49L-nCoV-S vaccine. A dose of 10^9^ PFU Sad23L-GFP and a dose of 10^9^ PFU Ad49L-GFP were used as sham control.

Five rhesus macaques aged 11–14 years (see Table S1) were first injected intramuscularly with a dose of 5 × 10^9^ PFU Sad23L-nCoV-S, and then at 4-week interval received a second dose of 5 × 10^9^ PFU Ad49L-nCoV-S vaccine. Three rhesus macaques aged 11–13 years were vaccinated by prime-boost regimen with a dose of 5 × 10^9^ PFU Sad23L-GFP and a dose of Ad49L-GFP vectorial adenoviruses as sham control.

### Enzyme-linked immunosorbent assay (ELISA)

The microtiter plates (Corning, USA) were coated overnight with 2 μg/ml of SARS-CoV-2 S (Sino Biological, 40589-V08B1) or RBD proteins (Sino Biological, 40592-V08H). Serum samples were twofold serially diluted and S or RBD binding antibody (S-BAb or RBD-BAb) were detected by ELISA. Secondary antibodies were goat anti-mouse IgG-HRP (Beijing Bersee Science and Technology, Co. Ltd, China), rabbit anti-monkey IgG-HRP (Bioss, China) and goat anti-human IgG-HRP conjugates (Sigma, USA), respectively. Endpoint titers were defined as the highest reciprocal serum dilution that yielded an absorbance >0.2 and a ratio of signal than cutoff (S/CO) >1. Log10 end point titers were reported [24].

Goat anti-mouse IgG1, IgG2a, IgG2b, or IgG3 heavy chain-HRP conjugates were used for IgG sub-classification according to manufacturer's instruction (Abcam, UK).

### Surrogate virus neutralization test (sVNT)

The surrogate virus based neutralization test (sVNT) was used for measuring neutralizing antibody (sNAb) to SARS-CoV-2 as previously described [25]. Briefly, microtiter plates were coated overnight with 2 μg/ml hACE2 protein (Sino Biological, China) at 4°C, followed by blocking with OptEIA assay diluent (BD). The HRP–RBD conjugate (3 ng) was pre-incubated with 100 μl of diluted serum samples for 1 h at 37°C, then added into hACE2 pre-coated plate for 1 h at room temperature. Plates were washed five times by PBST. A colorimetric signal was developed with TMB, and equal volume of stop solution was added to terminate the reaction. The absorbance reading was performed at 450 nm and 570 nm. Inhibition rate (%) = (1 − sample optical density value/negative control optical density value) × 100.

### Pseudovirus neutralization test (pVNT)

Pseudovirus expressing a luciferase reporter gene were generated for measuring of neutralizing antibody (pNAb) to SARS-CoV-2 as previously described [24]. Briefly, the packaging construct psPAX2 (Addgene), plasmid pLenti-CMV Puro-Luc (Addgene), and pcDNA3.1-SARS-CoV-2 SΔCT (deletion of the cytoplasmic tail) were co-transfected into HEK-293 T cells. The supernatants were collected 48 hours post-transfection and the pseudoviruses were purified by filtration through 0.45 µm filter. The pVNT titers (50% inhibitory concentration, IC_50_) were measured with HEK293T-hACE2 cells in 96-well tissue culture plates. Two-fold serial dilutions of heat-inactivated serum samples were prepared and mixed with 50 µl of pseudovirus. After incubation at 37°C for 1 h, the serum-virus mixture was added to HEK293T-hACE2 cells. Forty-eight hours after infection, cells were lysed in Steady-Glo Luciferase Assay (Promega) according to the manufacturer's instructions. The pVNT titer of SARS-CoV-2 antibody was defined as the sample dilution at which a 50% inhibition rate. Inhibition rate (%) = (1 − sample RLU/virus control RLU) × 100. Log10 pVNT (IC_50_) titer was reported.

### ELISpot

Monkey (or mouse) IFN-gamma, IL-2 or IL-4 ELISpotPLUS kits (MabTech) were used to determine SARS-CoV-2 S antigen-specific T lymphocyte response. Rhesus macaque's PBMCs (2 × 10^5^ cells/well) or mouse splenocytes (5 × 10^5^ cells/well) were stimulated with S peptides, S or RBD protein (5 μg/ml) in triplicates, respectively. Seventy-nine peptides encoding for amino acid sequence of SARS-CoV-2 S protein were predicted (http://www.iedb.org/) and synthesized by Guangzhou IGE Biotechnology LTD (Table S3). Spots were counted with a CTL Immunospot Reader (Cellular Technology Ltd). The results were expressed as spot forming cells (SFCs) per million cells.

### Intracellular cytokine staining (ICS) and flow cytometry

Mouse splenocytes (2 × 10^6^ cells/well) or monkey PBMCs (1 × 10^6^ cells/well) were stimulated with S peptide pools (3 μg/ml each peptide), or medium as negative control, in triplicates. After 4 h, the cells were incubated with Golgi Plug (BD) for 12 h at 37°C. Cells were collected and stained with anti-mouse or anti-monkey CD3, CD4 and CD8 surface marker antibodies (BD). Cells were fixed with IC fixation buffer, permeabilized with permeabilization buffer (BD) and stained with anti-mouse or anti-monkey interferon-γ (IFN-γ), interleukin-2 (IL-2), and tumour necrosis factor α (TNF-α) (BD). All samples were tested with BD FACS Canton flow cytometer (BD).

### Statistical analyses

Data are analysed with unpaired two-tailed *t* test, one-way ANOVA. Neutralizing antibody (NAb) titer data were log transformed before analysis. NAb titer data generated by the RBD-BAb and sVNT or S-BAb and pVNT assays were compared using Spearman nonparametric correlation. Statistically significant differences are indicated with asterisks (*P < 0.05; **P < 0.01 and ***P < 0.001). All graphs are generated with GraphPad Prism 7 software.

## Results

### Production and characterization of Sad23L-nCoV-S and Ad49L-nCoV-S vaccines

**Figure 1.**
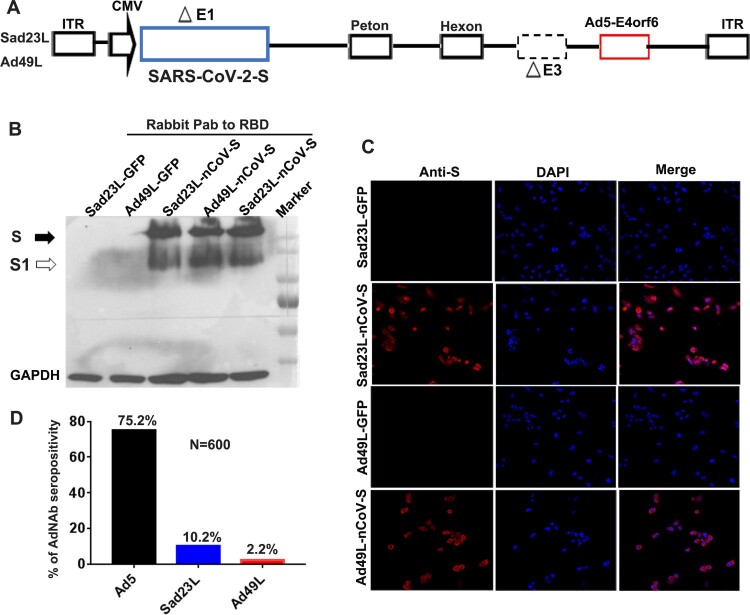
**Characterization of Sad23L-nCoV-S and Ad49L-nCoV-S vaccines.** (A) Recombinant adenovirus constructs Sad23L-nCoV-S and Ad49L-nCoV-S carrying the full-length S gene of SARS-CoV-2 under CMV promotor regulation within the deleted E1 region of Sad23L or Ad49L vector. (B) Western blot analysis for the expression of S protein from Sad23L-nCoV-S or Ad49L-nCoV-S infected HEK-293A cell lysates by rabbit polyclonal antibody to RBD. Sad23L-GFP or Ad49L-GFP virus infected cells were used as mock controls. (C) Expression of S protein in HEK-293A cells detected by immunofluorescence staining. (D) Seroprevalence of neutralizing antibody (AdNAb) to Ad5, Ad49L or Sad23L vector in 600 healthy blood donors.

An optimized and synthesized full-length S gene of SARS-CoV-2 was cloned into the deleted E1 region under cytomegalovirus (CMV) promotor regulation within two novel adenovirus vectorial Sad23L and Ad49L plasmids, designated as Sad23L-nCoV-S or Ad49L-nCoV-S, respectively (Figure 1A). The recombinant adenoviruses were rescued from HEK-293A. A large amount of Sad23L-nCoV-S and Ad49L-nCoV-S candidate vaccines were produced from HEK-293A cell cultures, and further purified and titrated over 10^11^ PFU/ml.

Expression of S protein was verified in Sad23L-nCoV-S and Ad49L-nCoV-S vaccine strains infected HEK-293A cells by Western blot with rabbit polyclonal antibodies to RBD, but not in the Sad23L-GFP and Ad49L-GFP viruses infected cells (Figure 1B). The expression of S protein in the vaccine strains-infected HEK-293A cells was also observed in red colour by an immunofluorescence staining, but not in the adenovirus-GFP infected control cells (Figure 1C). These results indicated that Sad23L-nCoV-S and Ad49L-nCoV-S vaccines could effectively produce SARS-CoV-2 S protein in the infected cells.

To measure pre-existing immunity to these two vectors, 600 healthy blood donor samples were collected from six cities crossing the south, north, east, west and central regions of China and were tested for neutralizing antibodies (AdNAb) reacting with Sad23L-GFP, Ad49L-GFP and Ad5-GFP viruses, respectively. The seroprevalence of Sad23L, Ad49L and Ad5 was 10.2%, 2.2%, or 75.2% respectively (Figure 1D), indicating that Sad23L and Ad49L vectors had low pre-exposure rate in the Chinese population.

### Animal tolerance of Sad23L-nCoV-S and Ad49L-nCoV-S vaccine inoculation

C57BL/6 mice (*n* = 5 per group) were intramuscularly injected with individual Sad23L-nCoV-S and Ad49L-nCoV-S vaccines at doses of 10^7^, 10^8^ and 10^9^ PFU, or with priming Sad23L-nCoV-S (10^9^ PFU) and boosting Ad49L-nCoV-S (10^9^ PFU) at 4 week post-priming, and adenovirus vectors and PBS as controls. Mice tolerated well the various doses of vaccines or vector controls at 28 or 56 days, presenting no obvious change of weight and body temperature when compared with injected PBS (see Figure S1A). In addition, histopathological examination of brain, lung, heart, liver, kidney, and muscle tissues (at intramuscular injection site and para-tissues) did not present significant pathological lesions over 4 weeks following the prime only or prime-boost injections (Figure S2).

Vaccination (*n* = 5) and sham control (*n* = 3) groups of rhesus macaques (Table S1) were first injected with 5×10^9^ PFU Sad23L-nCoV-S vaccine or Sad23L-GFP control, then boosting with a second dose of 5 × 10^9^ PFU Ad49L-nCoV-S vaccine or Ad49L-GFP control at 4 week post-priming, respectively. During the course of immunization, clinical parameters were monitored for eight weeks. All monkeys displayed normal appetite and mental state, and no obvious change of weight was observed with these eight animals, but body temperature had a sharp rise and recovered into 24 h (see Figure S1B). Hematological and biochemical examination of blood samples showed no notable variation when comparing the vaccinated or vector control animals during 8 weeks pre-vaccination and post-vaccination (see Figure S3 and Table S2).

### A single-shot immunization of Sad23L-nCoV-S or Ad49L-nCoV-S vaccine induced specific immune response in mice

**Figure 2.**
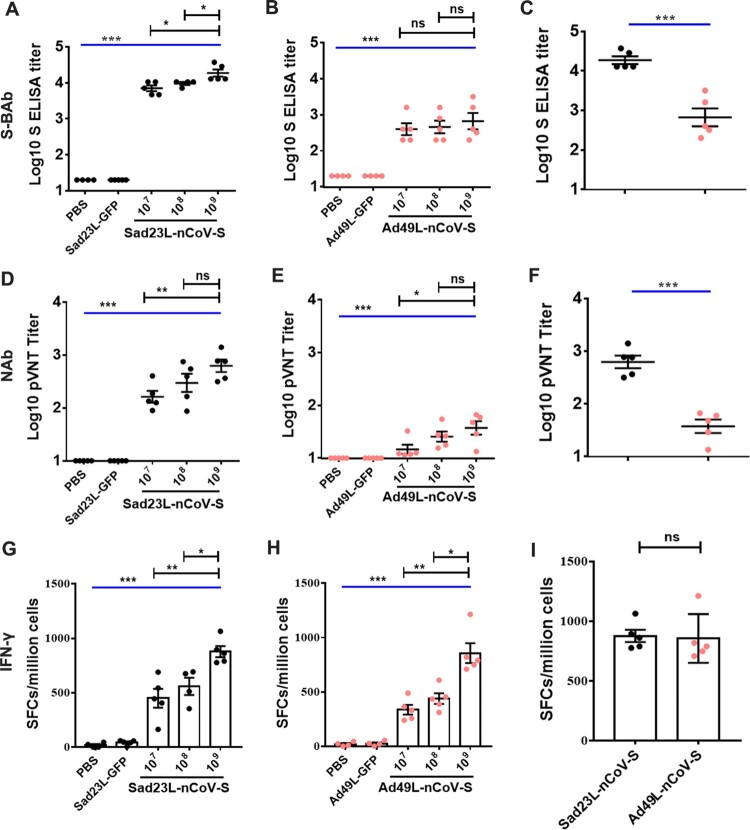
**Specific antibody and T-cell response of C57BL/6 mice inoculated with a single shot of Sad23L-nCoV-S or Ad49L-nCoV-S vaccine at three different doses.** C57BL/6 mice (*n* = 5/group) were immunized by a single dose of 10^7^, 10^8^ or 10^9^ PFU Sad23L-nCoV-S or Ad49L-nCoV-S vaccine. Mice sera and splenocytes were collected for measurement of antibody level and T-cell response 4 weeks post-immunization. (A–B) S binding antibody (S-BAb) titers were obtained by ELISA. (C) S-BAb was compared with Sad23L-nCoV-S and Ad49L-nCoV-S in 10^9^ PFU. (D–E) Neutralizing antibody (NAb) titers were obtained by pseudovirus-based neutralization test (pVNT). (F) pVNT was compared two vectors at the same doses of 10^9^ PFU. (G–H**)** IFN-γ secreting T-cell response (spot forming cells [SFCs]/million cells) of splenocytes to S peptides from Sad23L-nCoV-S or Ad49L-nCoV-S immunized mice was measured by ELISpot, respectively. (I) IFN-γ secreting T-cell response was compared two vectors at 10^9^ PFU. Data is shown as mean ± SEM (standard errors of means). *P* values are analysed by one-way ANOVA with twofold Bonferroni adjustment. Statistically significant differences are shown with asterisks (*, *P* < 0.05; **, *P* < 0.01 and ***, *P* < 0.001).

To evaluate the immunogenicity of individual Sad23L-nCoV-S and Ad49L-nCoV-S vaccines, a single dose of 10^7^, 10^8^ or 10^9^ PFU Sad23L-nCoV-S or Ad49L-nCoV-S vaccine was intramuscularly injected to C57BL/6 mice (*n* = 5/group). Vector control groups (*n* = 5/group) received 10^9^ PFU Sad23L-GFP or Ad49L-GFP viruses, and naïve control group (*n* = 5) received an equal volume of PBS, respectively. Four weeks post-immunization, titers of S binding antibody (S-BAb) were quantified by ELISA in a dose-dependent manner with both Sad23L-nCoV-S and Ad49L-nCoV-S immunized mice, but not in the control groups (*P* < 0.001, Figure 2A and B). A dose of 10^9^ PFU Sad23L-nCoV-S vaccine induced antibody titers of 10^4.27^ S-BAb, and higher titers than the same dose of Ad49L-nCoV-S vaccine with 10^3.02^ S-BAb (*P* < 0.001, Figure 2C). The neutralizing antibody (NAb) titers to SARS-CoV-2 were titrated in two individual vaccine immunized mice by pseudovirus-based NAb test (pVNT) at 50% inhibitory concentration (IC_50_), respectively (Figure 2D and E). A dose of 10^9^ PFU Sad23L-nCoV-S vaccine induced NAb titers of 10^2.79^ pVNT (IC_50_), and higher level than Ad49L-nCoV-S vaccine induced titers of 10^1.57^ pVNT (IC_50_) (*P* < 0.001, Figure 2F).

Specific T-cell response of isolated splenocytes was examined in vaccinated and sham mice after stimulation with S peptides (Figure 2G and H). A single dose of Sad23L-nCoV-S or Ad49L-nCoV-S vaccine elicited strong specific IFN-γ secreting T-cell response to S peptides (450.2–898.4 SFCs/million cells) and significantly higher than sham controls (*P* < 0.001, Figure 2G and H), and T-cell response of the two vectors vaccine at the same doses was not significantly different (*P* > 0.05, Figure 2I).

Overall, individual Sad23L-nCoV-S or Ad49L-nCoV-S vaccinated mice developed specific BAb and NAb antibodies and T-cell responses to S protein, RBD protein or S peptides of SARS-CoV-2 in a dose-dependent fashion, and Sad23L-nCoV-S induced higher antibodies titers than Ad49L-nCoV-S vaccine at the same dose (Figure 2), suggesting strong immunogenicity of the two novel adenovirus vectored COVID-19 vaccines.

### Prime-boost immunization of mice with Sad23L-nCoV-S and Ad49L-nCoV-S vaccines

**Figure 3.**
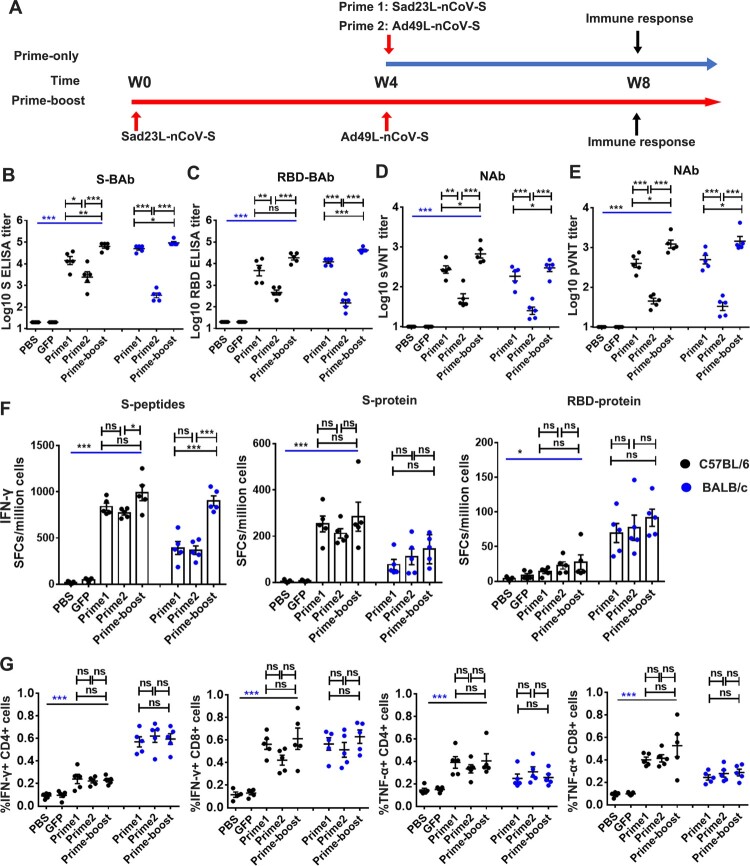
**Specific humoral and cellular immune response of C57BL/6 and BALB/c mice to prime-boost immunization with Sad23L-nCoV-S and Ad49L-nCoV-S vaccines.** (A) C57BL/6 and BALB/c mice (*n* = 5/group) were prime immunized with a dose of 10^9^ PFU Sad23L-nCoV-S vaccine and boosted with a dose of 10^9^ PFU Ad49L-nCoV-S vaccine at 4 week interval. Sera and splenocytes were collected from vaccinated or control mice for measurement of antibody and T-cell responses 4 weeks after both prime only and boosting immunizations. (B–C) Anti-S-BAb and RBD-BAb titers determined by ELISA. (D–E) NAb titers measured by sVNT and pVNT. (F) IFN-γ secreting T-cell response (SFCs/million cells) to S peptides, S or RBD protein measured by ELISpot. (G) Frequency of IFN-γ or TNF-α expressing CD4^+^ and CD8^+^ T-cell response to S peptides determined by ICS. Data are shown as a mean ± SEM. *P* values are analysed with one-way ANOVA and two-tailed *t* test. Statistically significant differences are shown with asterisks (*, *P* < 0.05; **, *P* < 0.01 and ***, *P* < 0.001).

To further improve reactivity and longevity of immune response to vaccines, the prime-boost vaccination regimen was utilized to immunize C57BL/6 and BALB/c mice (*n* = 5/group) with a prime dose of 10^9^ PFU Sad23L-nCoV-S on day 0 and a boost dose of 10^9^ PFU Ad49L-nCoV-S on day 28 (Figure 3A). In comparison with prime immunization with Sad23L-nCoV-S or Ad49L-nCoV-S, a booster with Ad49L-nCoV-S significantly increased BAb and NAb titers in mice (*P* < 0.05, Figure 3B–E). The S-BAb was 10^4.15^ (Prime 1: Sad23L-nCoV-S), 10^3.39^ (Prime 2: Ad49L-nCoV-S) and 10^4.81^ (Prime-boost: Sad23L-nCoV-S/Ad49L-nCoV-S) in C57BL/6, while S-BAb was 10^4.71^ (Prime 1), 10^2.54^ (Prime 2) and 10^4.96^ (Prime-boost) in BALB/c (Figure 3B); the RBD-BAb was 10^3.67^ (Prime 1), 10^2.67^ (Prime 2) and 10^4.26^ (Prime-boost) in C57BL/6, while RBD-BAb was 10^4.08^ (Prime 1), 10^2.41^ (Prime 2) and 10^4.62^ (Prime-boost) in BALB/c (Figure 3C); the sNAb titer of sVNT (IC_50_) was 10^2.43^ (Prime 1), 10^1.71^ (Prime 2) and 10^2.83^ (Prime-boost) in C57BL/6, while sNAb was 10^2.27^ (Prime 1), 10^1.40^ (Prime 2) and 10^2.48^ (Prime-boost) in BALB/c (Figure 3D); the pNAb titer of pVNT (IC_50_) was 10^2.61^ (Prime 1), 10^1.65^ (Prime 2) and 10^3.09^ (Prime-boost) in C57BL/6, while pNAb was 10^2.70^ (Prime 1), 10^1.52^ (Prime 2) and 10^3.16^ (Prime-boost) in BALB/c (Figure 3E), respectively. Profiling of IgG subclasses showed a predominant serum IgG2a to RBD protein associating with Th1 response in prime-boost vaccinated mice (Figure S4). Regarding specific T-cell response, the boosting with Ad49L-nCoV-S on Sad23L-nCoV-S prime immunization enhanced or stabilized specific IFN-γ-secretion T-cell responses to S peptides, S protein or RBD protein (Figure 3F), as well as levels of intracellular cytokines IFN-γ and TNF-α but not of IL-2 response to S peptides compared with single dose of vaccine vaccinated or sham control C57BL/6 and BALB/c mice (Figure 3G; Figure S5).

Taken together, the results suggest that prime-boost vaccination of two species of mice with Sad23L-nCoV-S followed by Ad49L-nCoV-S enhanced specific immune response to SARS-CoV-2 when compared with prime vaccination only with a single-shot of Sad23L-nCoV-S or Ad49L-nCoV-S vaccine.

### Rhesus macaques’ specific immune response to prime-boost vaccination with combined Sad23L-nCoV-S and Ad49L-nCoV-S vaccines

**Figure 4.**
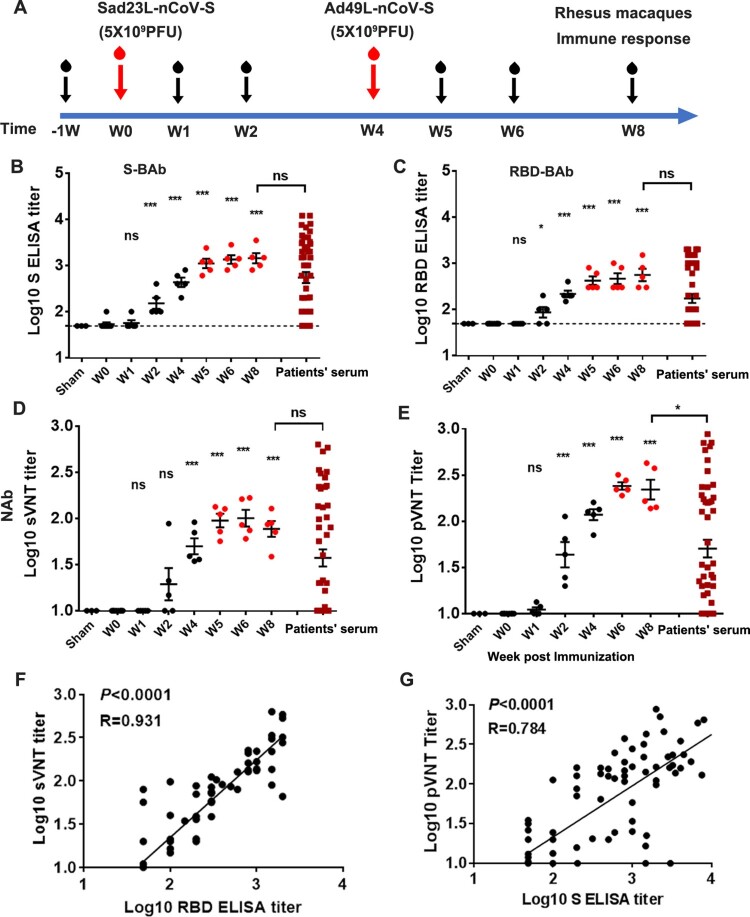
**Antibody reactivity of Rhesus macaques to prime-boost vaccination with Sad23L-nCoV-S and Ad49L-nCoV-S vaccines.** (A) Five rhesus macaques were prime immunized with 5 × 10^9^ PFU of Sad23L-nCoV-S vaccine and boosted with 5 × 10^9^ PFU of Ad49L-nCoV-S vaccine at 4 week interval. Blood samples were collected weekly from immunized or sham control macaques. Three macaques first immunized with 5 × 10^9^ PFU of Sad23L-GFP viruses and boosted with 5 × 10^9^ PFU of Ad49L-GFP viruses were used as sham controls. Convalescent serum samples from 25 asymptomatic, 14 mild and 9 severe COVID-19 infected patients were taken as positive controls. (B–C) S-BAb and RBD-BAb titers were tested by ELISA. (D–E) NAb titers were measured by sVNT and pVNT. (F–G) Correlation between RBD-BAb and sVNT or S-BAb and pVNT titers were compared using Spearman nonparametric correlation, respectively.

Eight rhesus macaques aged 11–14 years were selected and tested for the baseline values of antibody and T-cell response to SARS-CoV-2 S antigen from blood samples in pre-vaccination at week 1 or week 0 (Figure 4A; Table S1). The pre-existing AdNAb titer to Sad23L, Ad49L or Ad5 was detected < 1:10 in serum samples from all eight animals (see Table S1).

Five rhesus macaques were intramuscularly injected first with a dose of 5 × 10^9^ PFU Sad23L-nCoV-S, then with a boost injection of 5 × 10^9^ PFU Ad49L-nCoV-S vaccine 4 weeks later, while three sham macaque controls were injected with equal doses of Sad23L-GFP and Ad49L-GFP viruses, respectively (Figure 4A). Blood samples were collected weekly from these two groups of animals. Both BAb (S-BAb and RBD-BAb) and NAb (sNAb and pNAb) levels were titrated in the vaccinated group but not in sham group. BAb and NAb reactivity increased at week 2 post prime-immunization, at week 4 (W0 for boost-immunization), at weeks 5 and 6 and then stayed at high levels up to week 8 (Figure 4B–E). Titers of 10^3.16^ S-BAb (Figure 4B), 10^2.75^ RBD-BAb (Figure 4C), 10 ^2.01^ sNAb (sVNT IC_50_) (Figure 4D) and 10^2.38^ pNAb (pVNT IC_50_) (Figure 4E) were quantified and found higher than the corresponding antibody titers found in convalescent sera from 48 COVID-19 patients (*P* = 0.0384, Figure 4E), indicating obtaining protective efficacy against SARS-CoV-2 infection. Strong correlation between BAb and NAb titers was observed (*P* < 0.0001, *R* = 0.784–0.931; Figure 4F and G), suggesting the reliability of these antibody quantitative assays.

**Figure 5.**
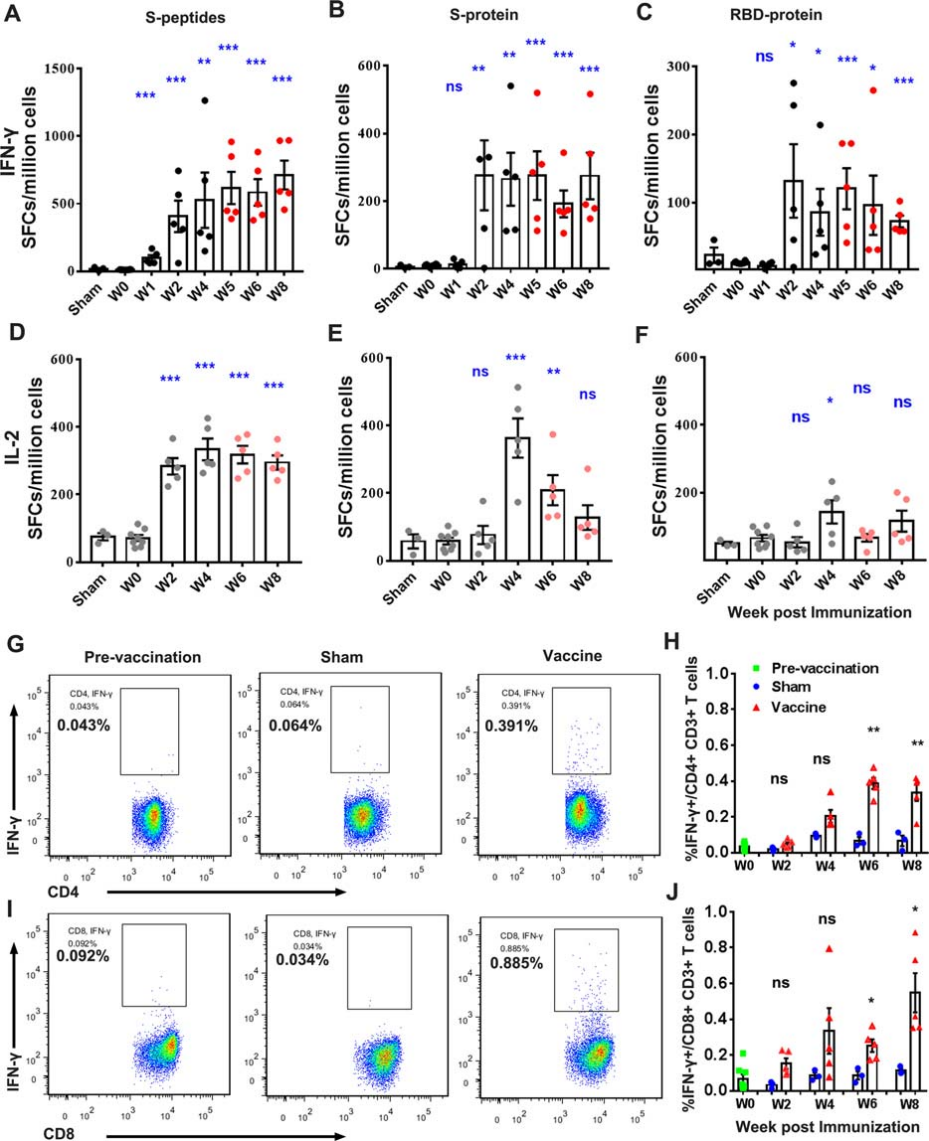
**Specific T-cell response of PBMCs from rhesus macaques immunized with Sad23L-nCoV-S and Ad49L-nCoV-S vaccines or sham controls by prime-boost vaccination regimen.** (A–C) IFN-γ or (D–F) IL-2 secreting T-cell response (SFCs/million cells) to S peptides, S or RBD protein was measured by ELISpot. (G–J) Frequency of intracellular IFN-γ expressing CD4^+^/CD8^+^ T-cell response to S peptides was determined by ICS, respectively. Data are shown as mean ± SEM. *P-*values are calculated with two-tailed *t* test. Statistically significant differences are shown with asterisks (*, *P* < 0.05; **, *P* < 0.01 and ***, *P* < 0.001).

PBMCs were isolated from whole blood of pre- and post-immunized monkeys for evaluation of T-cell responses to S peptides, S and RBD protein by ELISpot and ICS (Figure 5). Prime vaccination with a dose of Sad23L-nCoV-S vaccine induced an increase of IFN-γ secreting T-cell response to S peptides (406.6–526.3 SFCs/million cells) at weeks 2 and 4 post prime-vaccination, and then boosting with a dose of Ad49L-nCoV-S vaccines enhanced the IFN-γ secreting T-cell response (583.9–712.6 SFCs/million cells) at weeks 5–8 (Figure 5A). IFN-γ secreting T-cell reaction to S and RBD proteins stayed at high levels after prime-boost immunizations (Figure 5B and C). Relatively high IL-2 secreting T-cell response to S peptides, S and RBD proteins was observed (Figure 5D–F), but weak IL-4 secreting T-cell response by ELISpot (Figure S6). Frequency of intracellular IFN-γ expressing CD4^+^/CD8^+^ T-cell responses to S peptides was observed in vaccinated macaques at weeks 2–8 (Figure 5G–J), significantly higher than observed in pre-vaccination and sham controls (*P* < 0.05). Frequency of intracellular TNF-α expressing CD4^+^ T-cell response was also found significantly different between vaccinated and sham monkeys (*P* < 0.05; Figure S7A and B), but intracellular TNFα^+^ CD8^+^ or IL-2^+^ CD4^+^/CD8^+^ T-cell response to S peptides was not statistically different between groups (*P* > 0.05; Figure S7C-F).

In summary, prime-boost vaccination with Sad23L-nCoV-S and Ad49L-nCoV-S vaccines at an interval of 4 weeks elicited higher levels of specific antibody and T-cell responses against SARS-CoV-2 in rhesus macaques, which was recommended as COVID-19 vaccine candidates for clinical trials in humans.

### Duration of antibody response to Sad23L-nCoV-S and Ad49L-nCoV-S vaccines in mice and rhesus macaques

**Figure 6.**
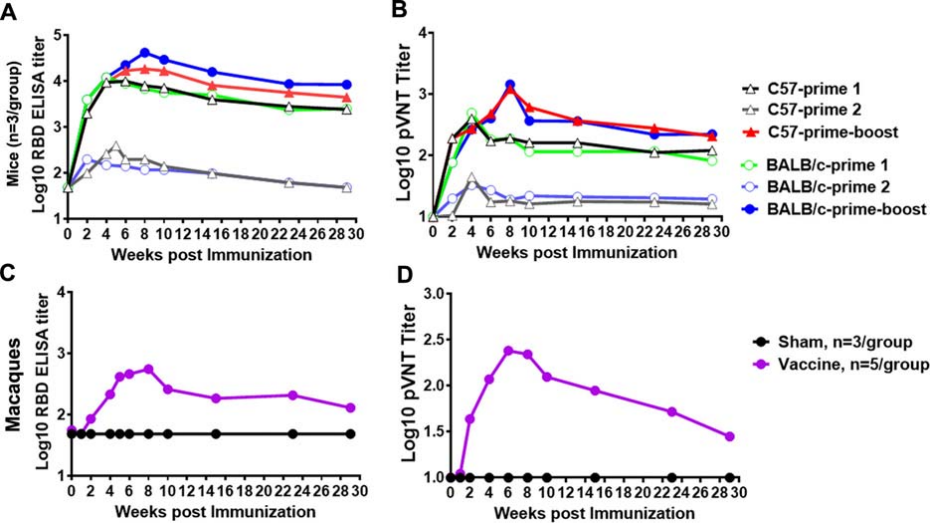
**Duration of antibody response of Sad23L-nCoV-S and Ad49L-nCoV-S vaccines in mice and Rhesus macaques.** (A) RBD-BAb and (B) pNAb were measured for 29 weeks in a single dose of Sad23L-nCoV-S (Prime 1) or Ad49L-nCoV-S (Prime 2), or prime-boost (Sad23L-nCoV-S and Ad49L-nCoV-S) vaccinated C57BL/6 and BALB/c mice. (C) RBD-BAb and (D) pNAb were measured for 29 weeks in prime-boost (Sad23L-nCoV-S/Ad49L-nCoV-S) vaccinated or sham (Sad23L-GFP/Ad49L-GFP) rhesus macaques. Mean Ab titers are presented in each group.

The follow-up detection of antibody response to the prime-boost vaccinated mice and macaques was carried out for 29 weeks after priming immunization of animals. The RBD-BAb and pNAb were slightly decreased and maintained at a higher level (>10^3.09^ or 10^2.32^) up to 7 months in the prime-boost (Sad23L-nCoV-S/Ad49L-nCoV-S) or a single dose of prime 1 (Sad23L-nCoV-S) immunized C57BL/6 and BALB/c mice (Figure 6A and B), while the RBD-BAb in vaccinated macaques presented a kinetic pattern similar in mice but the pNAb (pVNT IC_50_) were decreased gradually from high level of 10^2.38^ at 2 months to low level of 10^1.45^ at 7 months post prime-immunization (Figure 6C and D).

### The neutralizing antibody (AdNAb) response to adenoviral vectors in mice and rhesus macaques

**Figure 7.**
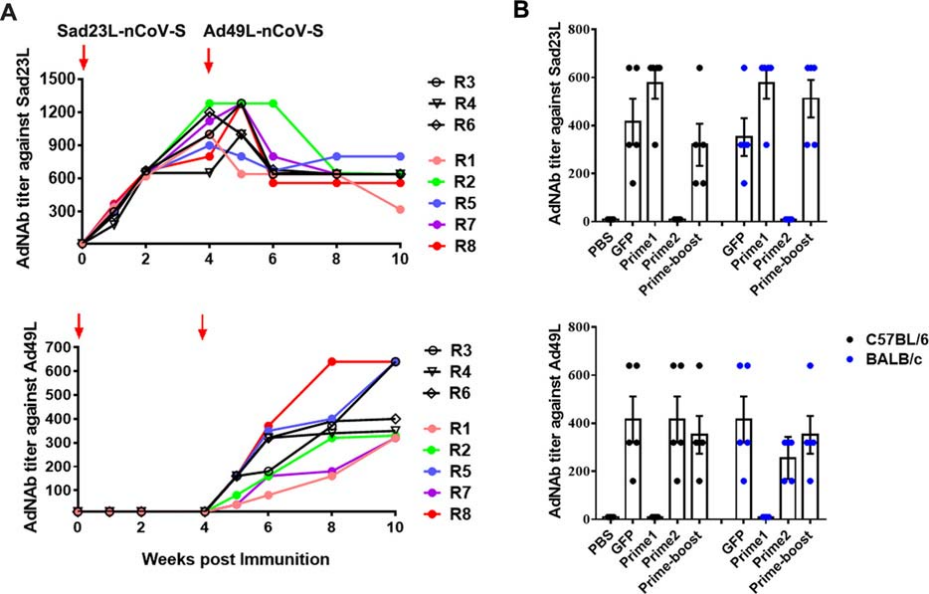
**AdNAb responses to Ad vectors.** Serum AdNAb titers to Sad23L and Ad49L vectors were measured in macaques (A) immunized by prime-boost inoculation or in C57BL/6 and BALB/c mice (B) 4 weeks post prime only or prime-boost vaccination with two vaccines or vectorial controls.

To test if the effectiveness of booster immunization correlated with neutralizing antibody (AdNAb) titer to the Ad vector, plasmas from mice and macaques were tested for AdNAb titers to Sad23L and Ad49L vectors (Figure 7). As expected after each immunization, AdNAb titers to the Ad vector were raised. AdNAb to Sad23L increased to high level after priming with Sad23L-nCoV-S, but did not neutralize Ad49L in macaques (Figure 7A) and mice (Figure 7B). A high AdNAb reactivity to an individual adenoviral vector of either Sad23L or Ad49L in macaques (AdNAb titer: 1:1280 or 1:640) and mice (1:576 or 1:352) was induced post prime immunization, but not enhanced post boost vaccination with a heterologous vector, which might limit the homologous boosting of adenovirus vectored vaccines (Figure 7). The results indicated that no cross-reactive AdNAb was elicited between Sad23L and Ad49L vector inoculated macaques and mice, and the effectiveness of booster immunizations with Ad49L-nCoV-S was not decreased.

## Discussion

A safe and effective COVID-19 vaccine is one of the most wanted goods in the world. Among 88 COVID-19 candidate vaccines in clinical trials (16 April 2021) [6], and some vaccines have been approved for emergency use [8–14, 16, 17, 26–30]. In the frontline of COVID-19 vaccine candidates in clinical trial phase III, the range of approaches includes four inactivated virus, three lipid nanoparticle (LNP)-encapsulated mRNA and four non-replicating adenovirus vectored vaccines [6]. Beyond general comments on the disadvantages of major types of COVID-19 vaccines [31, 32], the limitations of inactivated SARS-CoV-2 vaccines are crucial biosafety concerns regarding large amounts of infectious virus being processed above biological safety level-3 (BSL-3) condition and production capacity; for LNP-mRNA vaccines instability and safety need further consideration; for adenovirus vectored vaccines, pre-existing immunity to the carrier virus and the relative weakness of single-shot immunization appear limiting factors.

In this study, we generated two novel adenovirus vectored COVID-19 vaccines encoding the full-length S gene of SARS-CoV-2. The intact S glycoprotein rather than the shorter S or RBD proteins was shown to be the most effective antigen eliciting protective immunity against SARS-CoV-2 infection in DNA vaccines and Ad26 vectored vaccines [12, 13, 24]. The vectors Sad23L and Ad49L originated from simian adenovirus type 23 and human adenovirus type 49, respectively [21, 22], are novel adenoviral vector. Comparing Ad5-vectored COVID-19, ChAdox1 nCoV-19 and Ad26.COV2.S vaccines [8–12, 14, 17], three attractive aspects emphasized in this study are highlighted below.

Firstly, there is a low-seroprevalence of pre-existing antibodies to Sad23L and Ad49L vectors in humans. According to the investigation of AdNAb to three types of adenoviruses in Chinese population, the prevalence of both Sad23L and Ad49L AdNAb was below 10%, while the prevalence of Ad5 AdNAb was over 75% (Figure 1D). However, the pre-existing anti-Ad5 immunity might partly limit vaccine effectiveness, especially for populations aged over 50 [8, 9, 31, 32], while the low seroprevalence of antibodies to novel adenoviral vectors such as ChAdox1 [11], Ad26 [12, 14, 17, 33], and Sad23L and Ad49L used in this study might avoid a negative impact on vaccine efficacy [21, 22, 31].

Second, the prime-boost vaccination regimen with two heterologous adenoviruses vectored Sad23L-nCoV-S and Ad49L-nCoV-S vaccines examined in mice and macaques suggested that neutralizing antibody and specific IFN-γ secretion T-cell response were prolonged at high level after boosting vaccination (Figures 3–7). The boosting immune response to Ad49L-nCoV-S vaccine was not affected by the high AdNAb titer to Sad23L, and long-term sustained NAb to SARS-CoV-2 remained at high level over 29 weeks (Figure 6). Two recent publications compared prime only with a single-dose of ChAdOx1 nCoV-19 and homologous boost with a second dose of ChAdOx1 nCoV-19 vaccine in mice and pigs, and human clinical trials, showed that boost vaccination significantly increased the level of binding or neutralizing antibody response to SARS-CoV-2 [11, 16]. By boosting with Ad5-S vaccine to Ad26-S vaccine primed immunization, a higher level of immunity was observed in humans as reported in a recent Russian study [17]. Compared with homologous boosting of ChAdOx1 nCoV-19 or boosting of Ad5-S vaccines [11, 17], heterologous prime-boost vaccinations with Sad23L-nCoV-S and Ad49L-nCoV-S vaccines have the potential advantage of avoiding vector's immunity interfering with prime vaccination or enhancing pre-existing Ad5 immunity [21, 22, 31, 32].

Third, in order to achieve an effective vaccine immunity, a relatively low dose of two heterologous adenovirus vectored vaccines (10^9^ PFU, 2 × 10^10^ vp) with prime-boost regimen should theoretically reduce severe adverse reaction induced by a high dose of adenovirus vectored vaccine in clinical trials [8, 9, 11, 14]. In this study, a relatively low dose of Sad23L-nCoV-S and Ad49L-nCoV-S vaccines elicited a robust immunity in both mice and older rhesus macaques (aged 11–14 years), but no obvious clinical symptoms or histopathological changes were observed (see Figures S1–S3).

In addition, our results showed that a single intramuscular immunization with Sad23L-nCoV-S induces higher humoral immune responses than priming with Ad49L-nCoV-S in mice, while much less in terms of cellular immunity (Figures 2 and 3), and prime-boost groups significantly increased humoral immune responses than priming with Sad23L-nCoV-S or Ad49L-nCoV-S in mice (Figures 3B–E and 6), but did not increase in cellular response by boosting with Ad49L-nCoV-S (Figure 3F–G), except in BALB/c with S peptides (Figure 3F). This difference may be caused by the different serotype of adenovirus vectors (Sad23L and Ad49L were classified in species E and D of adenoviruses, respectively) [7], actual antigen or animals used in the study. The discrepancy was previously observed for the stronger T-cell response but weaker antibody reactivity from Ad4 and AdC68 vectored vaccines [34, 35]. The reason might be explained for that Ad49L induced the higher type I interferon (IFN) and down-regulated the transgene protein expression, and consequently stimulated a weak antibody response [34, 36]. However, the prime-boost vaccination with Sad23L-nCoV-S and Ad49L-nCoV-S vaccines indeed significantly increased the level of humoral immunity response to SARS-CoV-2 (Figure 6), and meanwhile the specific secreting and intracellular IFN-γ T-cell responses remained at high level after boosting vaccination (Figure 5).

Considering biosafety, sVNT and pVNT were used for replacing SARS-CoV-2 virus manipulation in this study, which were well demonstrated previously for a significant correlation with conventional live virus neutralization testing (cVNT) of NAb titers to SARS-CoV-2 (*P* < 0.0001, *R* = 0.7678–0.8591) [25]. This was also evidenced by close correlation between ELISA and pVNT or cVNT by other studies using adenovirus vectored COVID-19 vaccines in rhesus macaques (*P* < 0.0001, *R* = 0.8314–0.8427) [12], hamsters (*P* < 0.0001; *R* = 0.7849) [13] and human clinical phase II trial (*P* < 0.0001, *R* = 0.72–0.75) [9]. In addition, compared with BAb (10^2.73^ S-BAb or 10^2.24^ RBD-BAb) and NAb (10^1.59^ sNAb or 10^1.70^ pNAb) from a panel of convalescent serum samples from COVID-19 patients (Figure 4), the level of BAb (10^3.16^ S-BAb or 10^2.75^ RBD-BAb) and NAb (10^2.01^ sNAb or 10^2.38^ pNAb) from vaccinated macaques observed in this study was favorable and in line with other published data from adenovirus vectored COVID-19 vaccines [10, 12, 17], indicating the sufficient protection against SARS-CoV-2 infection. However, beyond the above indirect evidence for protective efficacy of vaccines, the lack of live virus injection challenging to vaccinated animals is a limitation to this work, which is planned for carrying out in future.

In conclusion, two novel adenovirus vectored COVID-19 vaccines were produced, when used in succession, safely elicit robust humoral and T-cell immune response to SARS-CoV-2 in mice and macaques. Prime-boost vaccination regimen with priming of Sad23L-nCoV-S and boosting of Ad49L-nCoV-S vaccines were recommended to develop the better protective immunity against SARS-CoV-2 in humans. These two vaccines are being planned for clinical phase I/II trials after an extensive safety evaluation has been carried out in pre-clinical animal examination.

## Supplementary Material

Clean_copy_of_supporting_material.docx
