# Supplementary material for: Prime-boost vaccination of mice and rhesus macaques with two novel adenovirus vectored COVID-19 vaccine candidates
Source: Emerg Microbes Infect. 2021 Jun 1;10(1):1002–15. doi: 10.1080/22221751.2021.1931466 (PMC8172228; doi:10.1080/22221751.2021.1931466)
Supplement: Clean_copy_of_supporting_material.docx [file TEMI_A_1931466_SM0275.docx]

**Supporting material**

**The PDF file includes**

Figure S1. Examination of weight and body temperature of mice and rhesus macaques inoculated with Sad23L-nCoV-S and Ad49L-nCoV-S vaccines.

Figure S2. Histopathological examination from Sad23L-nCoV-S and Ad49L-nCoV-S vaccines inoculated C57BL/6 mice

Figure S3. Kinetic change of hematological and clinical biochemistry indexes during the course of vaccinated or sham control rhesus macaques

Figure S4. IgG subclass antibodies against RBD protein in sera of C57BL/6 and BALB/c mice immunized by prime only or prime-boost with Sad23L-nCoV-S and Ad49L-nCoV-S vaccines

Figure S5. Frequency of IL-2 expressing CD4+/CD8+ T cell responses of splenocytes from prime-boost immunized C57BL/6 and BALB/c mice with Sad23L-nCoV-S and Ad49L-nCoV-S vaccines

Figure S6. IL-4 secreting T cell response in prime-boost immunized rhesus macaques with Sad23L-nCoV-S and Ad49L-nCoV-S vaccines

Figure S7. Frequency of intracellular TNFα and IL-2 expressing T cell response in PBMCs from rhesus macaques immunized with Sad23L-nCoV-S and Ad49L-nCoV-S vaccines or sham controls

Table S1. Basic information for rhesus macaques pre-vaccination

Table S2. Measuring of hematological and biochemistry indexes of rhesus macaques in the course of pre- and post-vaccination with Sad23L-nCoV-S and Ad49L-nCoV-S vaccines

Table S3. Peptides derived from amino acid sequences of SARS-CoV-2 S protein used

in ELISpot and ICS


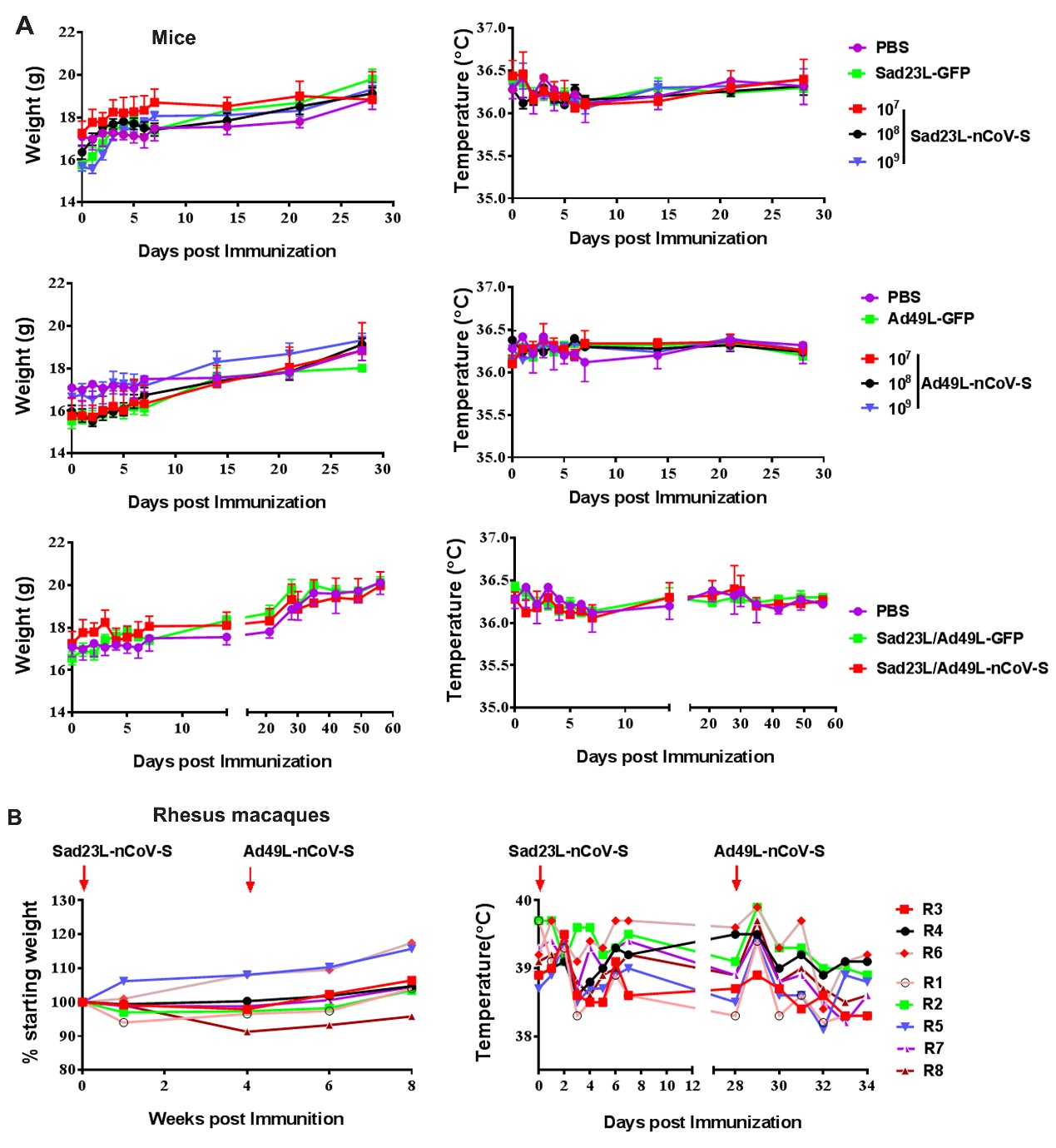


**Figure S1.** **Examination of weight and body temperature of mice and rhesus macaques inoculated with Sad23L-nCoV-S and Ad49L-nCoV-S vaccines.** Animals were intramuscularly immunized by prime only with either Sad23L-nCoV-S or Ad49LnCoV-S vaccine at three different doses, or by prime-boost with first Sad23L-nCoV-S and boost with Ad49L-nCoV-S vaccines at 4 week interval. Body weight and temperature were monitored during the course up to 60 days for mice or 8 weeks for monkeys. (A) C57BL/6 mice (n=5/group) immunized with a dose of 10^7^, 10^8^ or 10^9^ PFU Sad23L-nCoV-S vaccine, 10^9^ PFU Sad23L-GFP and an equal volume of PBS controls (top panel); a dose of 10^7^, 10^8^ or 10^9^ PFU Ad49L-nCoV-S vaccine, 10^9^ PFU Ad49L-GFP and an equal volume of PBS controls (middle panel); a dose of 10^9^ PFU

Sad23L-nCoV-S followed by a dose of 10^9^ PFU Ad49L-nCoV-S, or 10^9^ PFU Sad23LGFP and 10^9^ PFU Ad49L-GFP vectorial viruses and an equal volume of PBS controls

(low panel). (B) Rhesus monkeys (11-14y) inoculated with a dose of 5×10^9^ PFU Sad23L-nCoV-S and a dose of 5×10^9^ PFU Ad49L-nCoV-S vaccines (R1, R2, R5, R7 and R8), or 5×10^9^ PFU Sad23L-GFP and a dose of 5×10^9^ PFU Ad49L-GFP controls

(R3, R4 and R6).


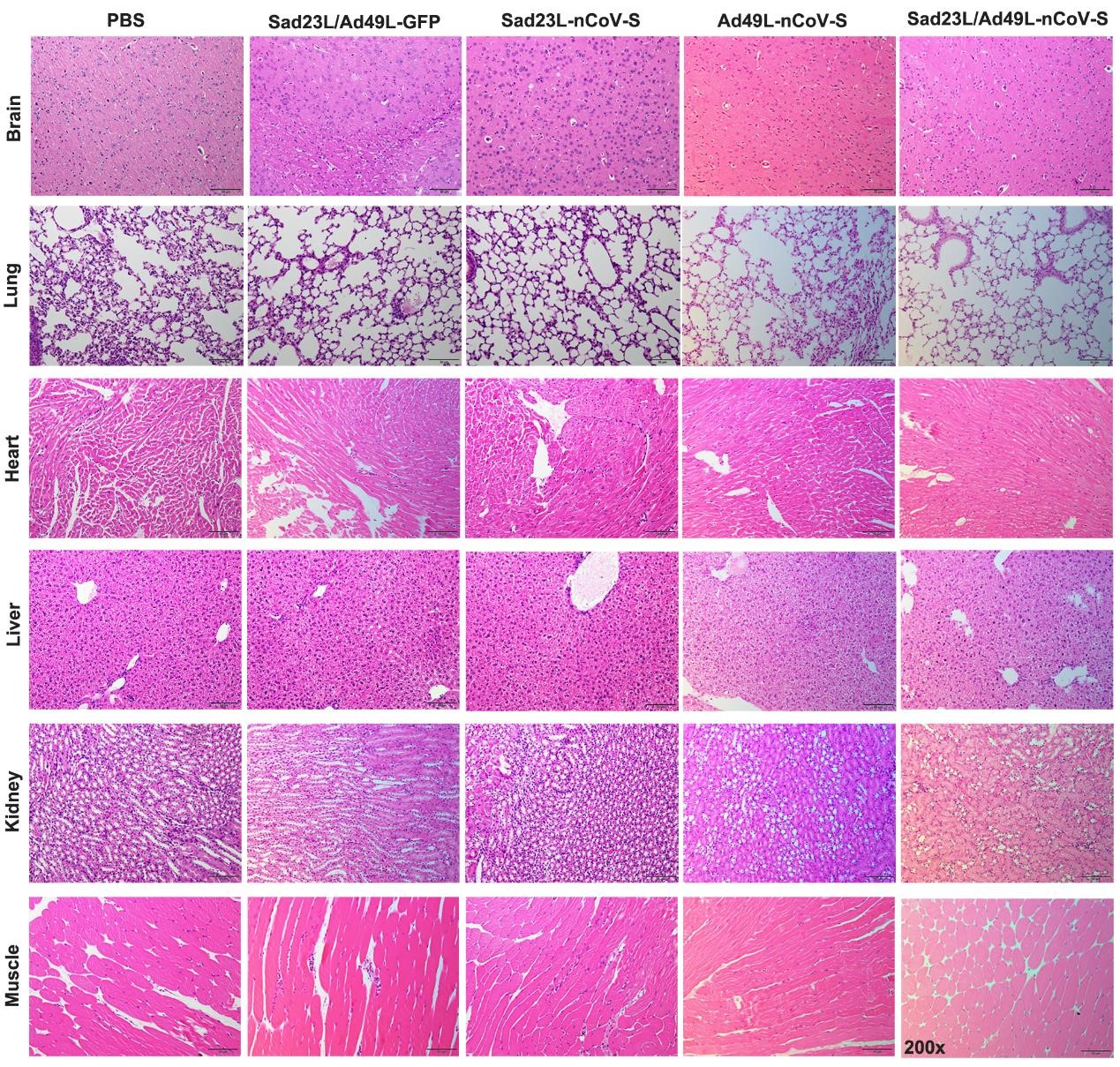


**Figure S2. Histopathological examination from Sad23L-nCoV-S and Ad49LnCoV-S vaccines inoculated C57BL/6 mice.** Histopathological examination was carried out for brain, lung, heart, liver, kidney and muscle tissues (at intramuscular injection site and para-tissues) of mice in 4 weeks post prime only (10^9^ PFU) or primeboost (10^9^ PFU) immunization with these two vaccines. Sad23L/Ad49L-GFP group was primed immunized with 10^9^ PFU Sad23L-GFP and boosted with 10^9^ PFU Ad49L-

GFP at 4 weeks post-priming. Tissues were stained with hematoxylin and eosin.


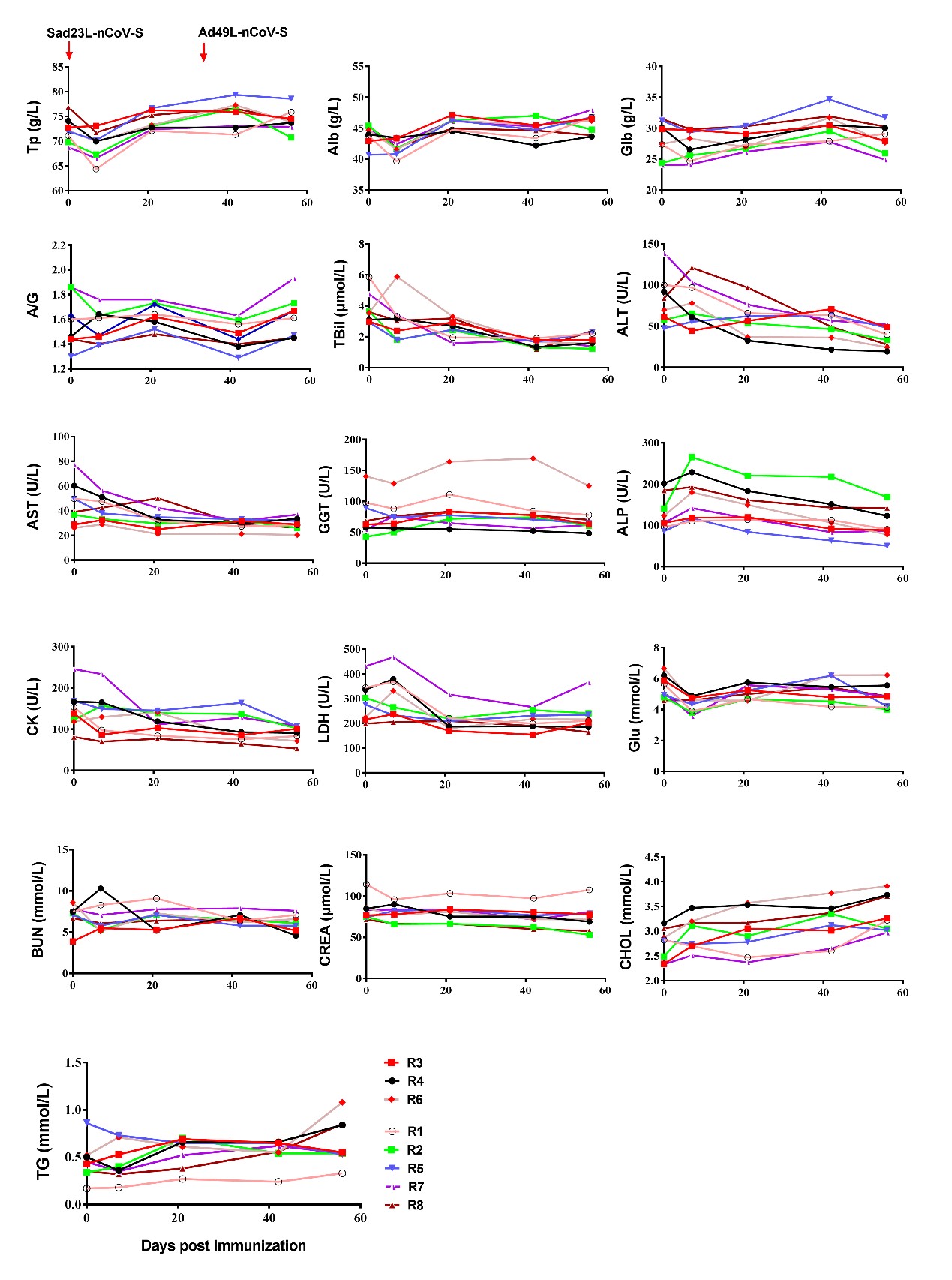


**Figure S3. Kinetic change of hematological and clinical biochemistry indexes during the course of vaccinated or sham control rhesus macaques.** Rhesus macaques were intramuscularly inoculated by prime-boost immunization with Sad23LnCoV-S and Ad49L-nCoV-S vaccines at an interval of 4 weeks. A panel of hematological and biochemical indexes were measured from blood samples at different time points. TP, Total protein; Alb, Albumin; Glb, Globulin; A/G, Albumin/globulin ratio; TBil, Total bilirubin; ALT, Alanine aminotransferase; AST, Aspartate aminotransferase; GGT, γ –glutamyltranspeptidase; ALP, Alkaline phosphatase; CK, Creatine kinase; LDH, Lactate dehydrogenase; Glu, Glucose; BUN, Blood urea nitrogen; CREA, Creatinine; CHOL, Total cholesterol; TG, Triglycerides. R1-R8

indicate rhesus macaques.


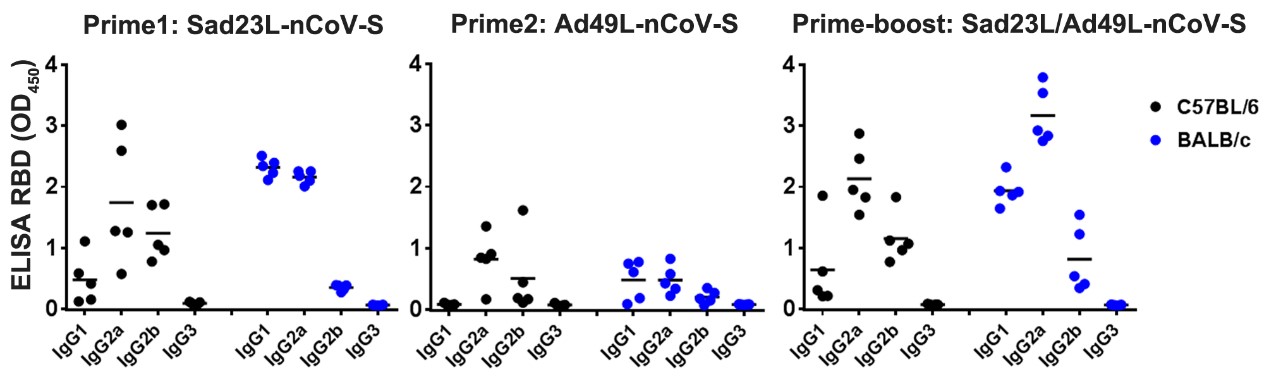


**Figure S4. IgG subclass antibodies against RBD protein in sera of C57BL/6 and BALB/c mice immunized by prime only or prime-boost with Sad23L-nCoV-S and Ad49L-nCoV-S vaccines.** The sera were collected 4 weeks post prime only or primeboost immunized C57BL/6 and BALB/c mice. IgG subclass antibodies in sera were detected against RBD protein by ELISA with secondary antibody-HRP conjugate specific to mouse IgG subclass.


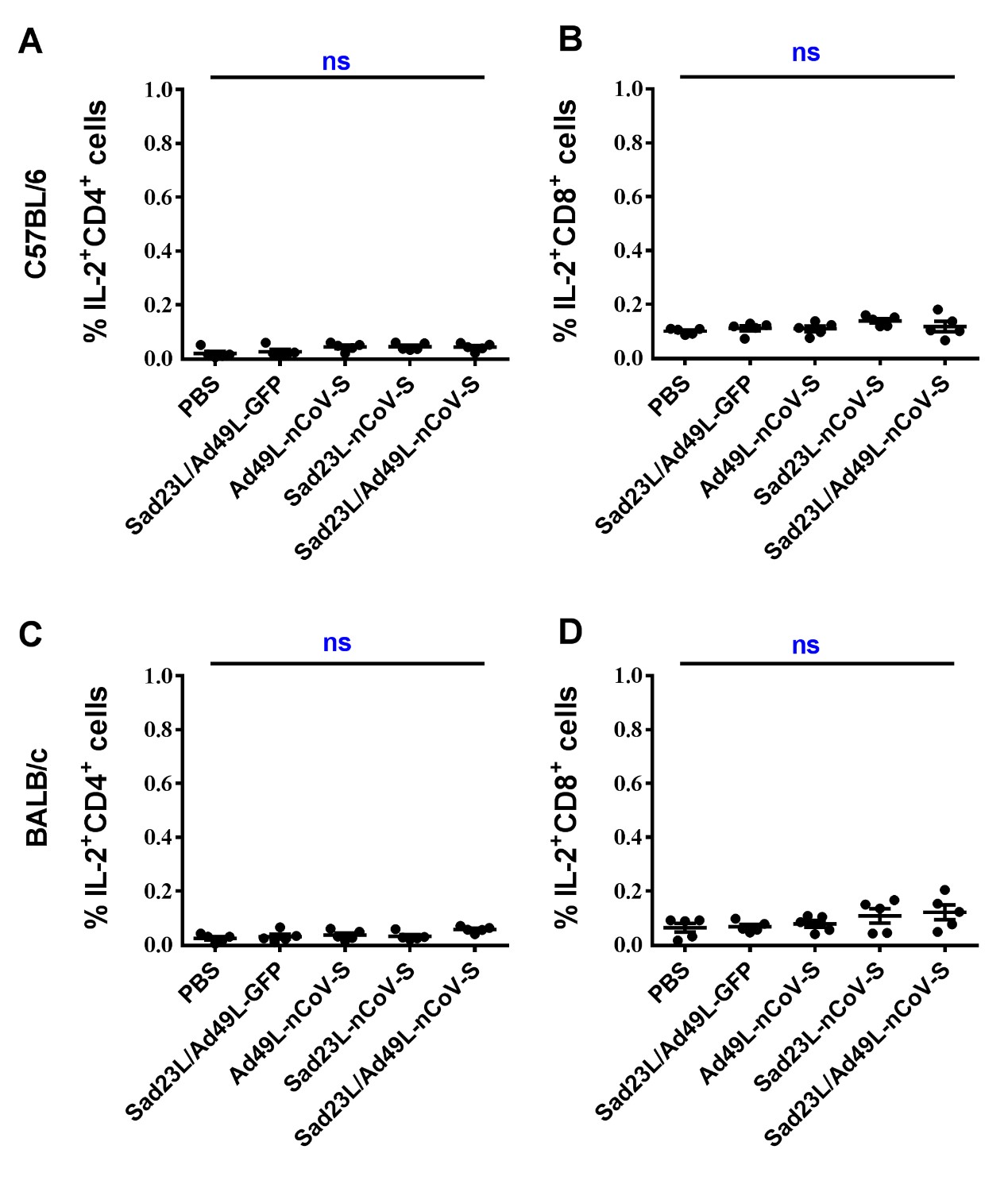


**Figure S5. Frequency of IL-2 expressing CD4+/CD8+ T cell responses of splenocytes from prime-boost immunized C57BL/6 and BALB/c mice with Sad23L-nCoV-S and Ad49L-nCoV-S vaccines.** (**A** and **B**) Frequency of IL-2^+^ CD4^+^ or CD8^+^ T cell responses of splenocytes to S peptides from C57BL/6, or (**C** and **D**) from BALB/c mice. Data were shown as means ± SEM (standard errors of means). *P* values were analyzed by one-way ANOVA. ns, *P*>0.05 and no significant difference.


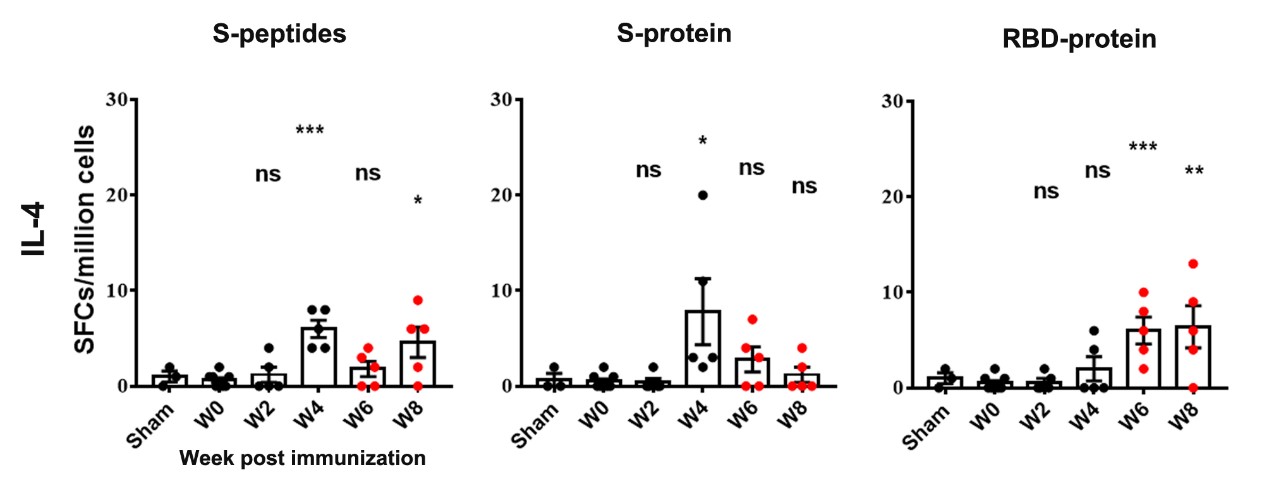


# Figure S6. IL-4 secreting T cell response in prime-boost immunized rhesus macaques with Sad23L-nCoV-S and Ad49L-nCoV-S vaccines. The number of

specific IL-4 secreting T cells (SFCs/million cells) to S peptides, S or RBD protein in PBMCs of monkeys was measured by ELISpot, respectively. Data were shown as means ± SEM (standard errors of means). *P* values were analyzed by one-way ANOVA with 2-fold Bonferroni’s test. Statically significant differences were showed with asterisks (*, *P*<0.05; **, *P*< 0.01 and ***, *P*< 0.001). ns, *P*>0.05 and no significant difference.


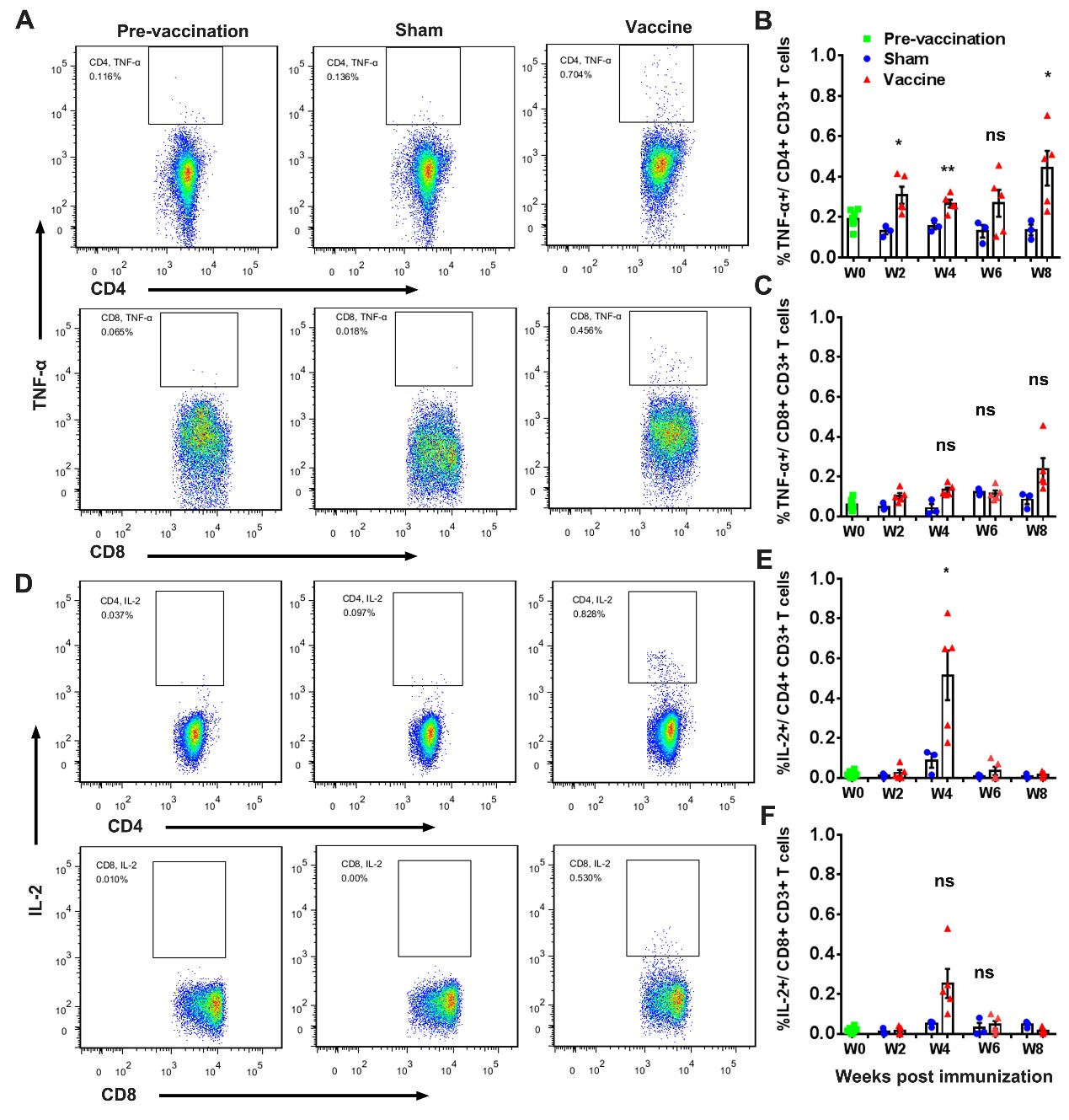


**Figure S7. Frequency of intracellular TNFα and IL-2 expressing** **T cell response in PBMCs from rhesus macaques immunized with Sad23L-nCoV-S and Ad49LnCoV-S vaccines or sham controls.** (A-C) Frequency of TNF-α^+^ CD4^+^/CD8^+^ T cell response to S peptides. (D-F) Frequency of IL-2^+^ CD4^+^/CD8^+^ T cell response to S peptides. Data were shown as means ± SEM (standard errors of means). *P* values were analyzed by Student's *t* test. Statically significant differences were showed with asterisks (*, *P*<0.05; **, *P*< 0.01 and ***, *P*< 0.001). ns, *P*>0.05 and no significant difference.

# Table S1. Basic information of rhesus macaques pre-vaccination

| Group | ID | Macaques  (gender) | Age  (year) | Weight  (kg) | AdNAb titer of pre-exposure to  Ad5 Sad23L Ad49L | | |
| --- | --- | --- | --- | --- | --- | --- | --- |
| Sham | 00159 | R3(M) | 13 | 11.79 | <1:10 | <1:10 | <1:10 |
|  | 06120011 | R4(M) | 13 | 6.76 | <1:10 | <1:10 | <1:10 |
|  | 08050591 | R6(M) | 12 | 7.99 | <1:10 | <1:10 | <1:10 |
| Vaccination | 01191 | R1 (M) | 14 | 11.56 | <1:10 | <1:10 | <1:10 |
|  | 01399 | R2(M) | 13 | 7.51 | <1:10 | <1:10 | <1:10 |
|  | 00943 | R5(M) | 13 | 9.32 | <1:10 | <1:10 | <1:10 |
|  | 00807 | R7(M) | 12 | 10.81 | <1:10 | <1:10 | <1:10 |
|  | 08040661 | R8 (M) | 11 | 8.72 | <1:10 | <1:10 | <1:10 |

# Table S2. Measuring of hematological and biochemistry indexes of rhesus macaques in the course of pre- and post-vaccination with Sad23L-nCoV-S and Ad49L-nCoV-S vaccines


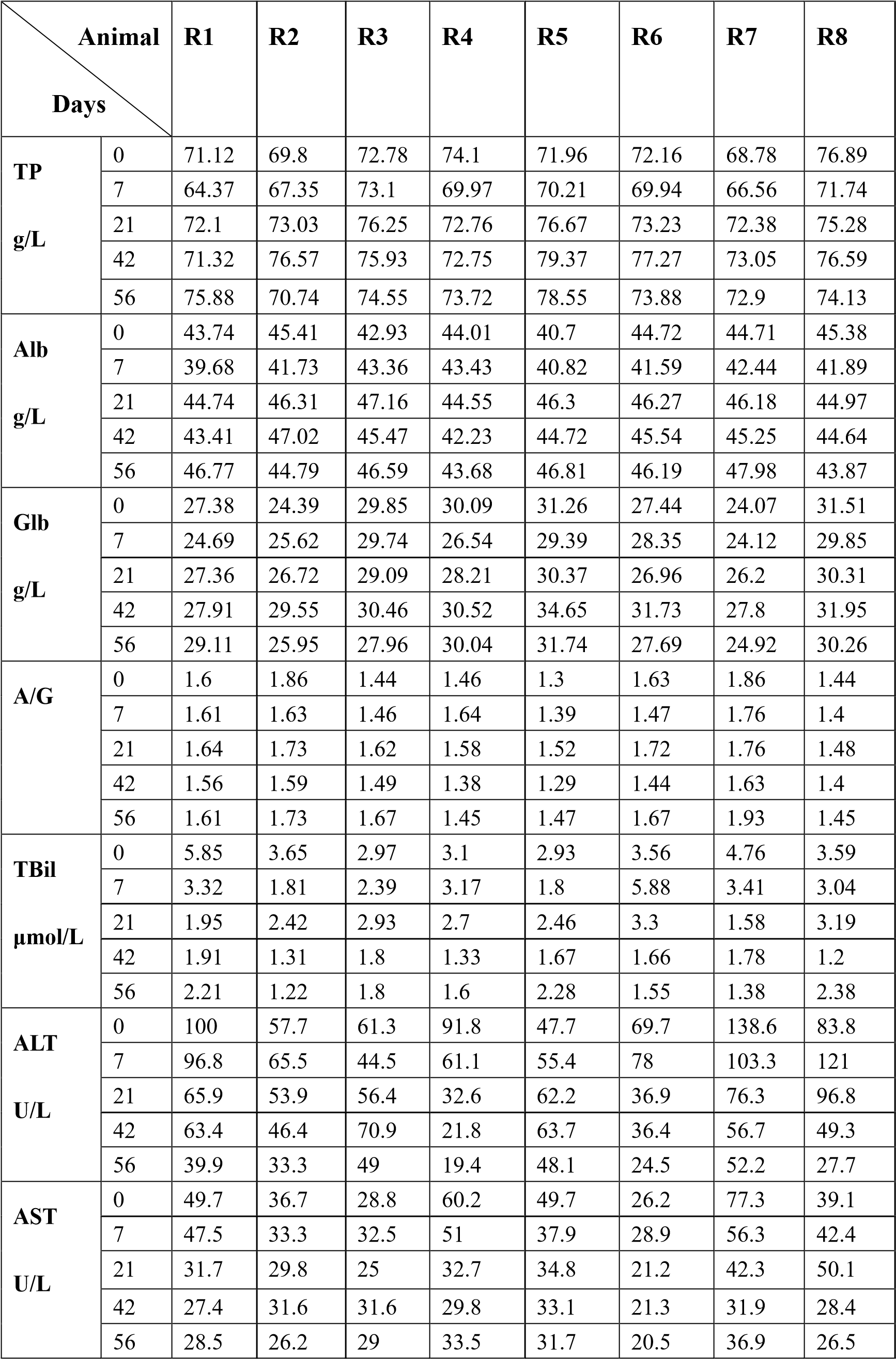


| **GGT**  **U/L** | 0 | 97.9 | 42.7 | 62.7 | 57.6 | 89.2 | 140.5 | 54.5 | 68.3 |
| --- | --- | --- | --- | --- | --- | --- | --- | --- | --- |
|  | 7 | 88 | 50.1 | 64.3 | 56.3 | 74.5 | 128.8 | 75.9 | 76 |
|  | 21 | 110.9 | 71.8 | 83.2 | 55 | 77.7 | 164 | 64.9 | 83.5 |
|  | 42 | 84.6 | 74.2 | 77.6 | 52.2 | 70.8 | 169.4 | 56.6 | 77.8 |
|  | 56 | 78.2 | 60.6 | 63.3 | 48.3 | 64.7 | 125 | 61.8 | 70.1 |
| **ALP**  **U/L** | 0 | 95.85 | 141.27 | 105.9 | 201.57 | 85.65 | 123.09 | 106.01 | 184.39 |
|  | 7 | 110.17 | 265.49 | 118.37 | 229.04 | 117.7 | 179.86 | 142.02 | 193.16 |
|  | 21 | 113.51 | 220.7 | 119.59 | 183.41 | 83.99 | 149.54 | 116.65 | 161.14 |
|  | 42 | 112.95 | 217.72 | 91.87 | 151.17 | 63.11 | 106.35 | 83.32 | 142.87 |
|  | 56 | 90.59 | 168.74 | 88.32 | 122.93 | 50.26 | 77.8 | 86.85 | 142.28 |
| **CK**  **U/L** | 0 | 153.4 | 122.7 | 138.3 | 166.7 | 168.6 | 118.5 | 245.5 | 81.4 |
|  | 7 | 97.8 | 156.8 | 87 | 165.1 | 149.2 | 129.6 | 233.5 | 70.1 |
|  | 21 | 84.1 | 139 | 103.1 | 118.9 | 145.3 | 140.5 | 111.7 | 76.7 |
|  | 42 | 75.6 | 136.6 | 86 | 93 | 163.9 | 81.3 | 128 | 64.9 |
|  | 56 | 83.6 | 101.9 | 101.1 | 90.9 | 107.1 | 71.5 | 108.2 | 53 |
| **LDH**  **U/L** | 0 | 344.2 | 302.9 | 215.7 | 336.3 | 275.3 | 224.2 | 430.9 | 198.9 |
|  | 7 | 368.7 | 265.6 | 237.2 | 378.8 | 233.4 | 331.6 | 467.2 | 205.6 |
|  | 21 | 220.9 | 219.2 | 170.1 | 187.3 | 209.2 | 180.1 | 316.6 | 209.6 |
|  | 42 | 199.4 | 253.9 | 155 | 188.5 | 231.2 | 217.7 | 266 | 189.9 |
|  | 56 | 210.4 | 241.1 | 202.5 | 185.8 | 235 | 216.5 | 365.4 | 164.7 |
| **GLU mmol/L** | 0 | 5.63 | 4.79 | 5.88 | 6.2 | 4.94 | 6.66 | 5.11 | 4.59 |
|  | 7 | 3.9 | 3.81 | 4.75 | 4.87 | 4.34 | 4.59 | 3.58 | 4.6 |
|  | 21 | 4.67 | 4.66 | 5.25 | 5.75 | 5.24 | 4.58 | 5.57 | 5 |
|  | 42 | 4.17 | 4.52 | 4.79 | 5.46 | 6.19 | 6.22 | 5.31 | 5.42 |
|  | 56 | 4.14 | 4 | 4.83 | 5.56 | 4.2 | 6.23 | 4.85 | 4.87 |
| **BUN mmol/L** | 0 | 7.5 | 7.3 | 3.9 | 7.5 | 7.1 | 8.6 | 7.8 | 6.7 |
|  | 7 | 8.3 | 5.3 | 5.5 | 10.3 | 5.8 | 5.1 | 7.1 | 6.1 |
|  | 21 | 9.1 | 7.1 | 5.3 | 5.2 | 7.1 | 7.3 | 7.8 | 6.4 |
|  | 42 | 6.4 | 6.5 | 6.7 | 7.1 | 5.8 | 6.2 | 7.9 | 6.6 |
|  | 56 | 7.1 | 6.1 | 5.2 | 4.6 | 5.8 | 6.6 | 7.6 | 6.1 |
| **CREA**  **μmol/L** | 0 | 114.3 | 74.9 | 76.6 | 84.7 | 74.2 | 85.4 | 82.9 | 71.4 |
|  | 7 | 95.9 | 65.9 | 77.8 | 90.1 | 82.1 | 77 | 83.6 | 66.8 |
|  | 21 | 103.3 | 66.5 | 83.7 | 75.2 | 83.6 | 81.1 | 84 | 66.5 |
|  | 42 | 97.3 | 62.6 | 80.5 | 75.2 | 76.6 | 72.1 | 71.4 | 59.9 |
|  | 56 | 107.5 | 53 | 78 | 68.9 | 78.9 | 71.6 | 80.8 | 57.7 |
| **CHOL mmol/L** | 0 | 2.82 | 2.49 | 2.34 | 3.16 | 2.81 | 2.87 | 2.33 | 3.05 |
|  | 7 | 2.7 | 3.11 | 2.7 | 3.47 | 2.74 | 3.2 | 2.51 | 3.16 |
|  | 21 | 2.47 | 2.9 | 3.05 | 3.53 | 2.78 | 3.57 | 2.37 | 3.17 |
|  | 42 | 2.6 | 3.35 | 3.01 | 3.46 | 3.12 | 3.77 | 2.65 | 3.37 |
|  | 56 | 3.22 | 3.05 | 3.26 | 3.73 | 3.02 | 3.91 | 2.97 | 3.71 |
| **TG** | 0 | 0.17 | 0.34 | 0.43 | 0.5 | 0.86 | 0.52 | 0.45 | 0.35 |
|  | 7 | 0.18 | 0.4 | 0.53 | 0.36 | 0.73 | 0.71 | 0.35 | 0.32 |
| **mmol/L** | 21 | 0.27 | 0.7 | 0.69 | 0.66 | 0.65 | 0.61 | 0.52 | 0.38 |
|  | 42 | 0.24 | 0.54 | 0.65 | 0.66 | 0.65 | 0.55 | 0.62 | 0.56 |
|  | 56 | 0.33 | 0.54 | 0.55 | 0.84 | 0.53 | 1.08 | 0.54 | 0.85 |

**Table S3. Peptides derived from amino acid sequences of SARS-CoV-2 S protein used in ELISpot and ICS**_._

| Peptides | Sequence | Peptides | Sequence |
| --- | --- | --- | --- |
| 1 | SSVLHSTQDLFLPF | 41 | TTRTQLPPAYTNSF |
| 2 | FLGVYYHKNNKSWM | 42 | HTPINLVRDLPQGF |
| 3 | FLPFFSNVTWFHAI | 43 | TTAPAICHDGKAHF |
| 4 | QGFSALEPLVDLPI | 44 | KTPPIKDFGGFNFS |
| 5 | KTQSLLIVNNATNV | 45 | TTDAVRDPQTLEIL |
| 6 | REFVFKNIDGYFKI | 46 | MSFPQSAPHGVVFL |
| 7 | AAYYVGYLQPRTFL | 47 | LTPTWRVYSTGSNV |
| 8 | FQFCNDPFLGVYYH | 48 | LTDEMIAQYTSALL |
| 9 | VSSQCVNLTTRTQL | 49 | YSNNSIAIPTNFTI |
| 10 | RVYSSANNCTFEYV | 50 | TITSGWTFGAGAAL |
| 11 | VSGTNGTKRFDNPV | 51 | LTESNKKFLPFQQF |
| 12 | VVIGIVNNTVYDPL | 52 | SALLAGTITSGWTF |
| 13 | QVAVLYQDVNCTEV | 53 | ILPDPSKPSKRSFI |
| 14 | SSVLNDILSRLDKV | 54 | FTRGVYYPDKVFRS |
| 15 | FIAGLIAIVMVTIM | 55 | STPCNGVEGFNCYF |
| 16 | YQTSNFRVQPTESI | 56 | CVADYSVLYNSASF |
| 17 | AENSVAYSNNSIAI | 57 | DLCFTNVYADSFVI |
| 18 | RSFIEDLLFNKVTL | 58 | YQPYRVVVLSFELL |
| 19 | AIPTNFTISVTTEI | 59 | FPNITNLCPFGEVF |
| 20 | SIVRFPNITNLCPF | 60 | NNLDSKVGGNYNYL |
| 21 | SAPHGVVFLHVTYV | 61 | YLYRLFRKSNLKPF |
| 22 | VAYSNNSIAIPTNF | 62 | DSKVGGNYNYLYRL |
| 23 | FAMQMAYRFNGIGV | 63 | DYSVLYNSASFSTF |
| 24 | LIAIVMVTIMLCCM | 64 | CYGVSPTKLNDLCF |
| 25 | TGIAVEQDKNTQEV | 65 | YGFQPTNGVGYQPY |
| 26 | VFAQVKQIYKTPPI | 66 | RKRISNCVADYSVL |
| 27 | VTYVPAQEKNFTTA | 67 | TRFASVYAWNRKRI |
| 28 | NTLVKQLSSNFGAI | 68 | DFTGCVIAWNSNNL |
| 29 | FQPTNGVGYQPYRV | 69 | SVLYNSASFSTFKC |
| 30 | QDVVNQNAQALNTL | 70 | VSPTKLNDLCFTNV |
| 31 | ARSVASQSIIAYTM | 71 | IAPGQTGKIADYNY |
| 32 | CAQKFNGLTVLPPL | 72 | FKCYGVSPTKLNDL |
| 33 | GVTQNVLYENQKLI | 73 | YSVLYNSASFSTFK |
| 34 | ENQKLIANQFNSAI | 74 | SGINASVVNIQKEI |
| 35 | AEHVNNSYECDIPI | 75 | IAGLIAIVMVTIML |
| 36 | TFGAGAALQIPFAM | 76 | AKNLNESLIDLQEL |
| 37 | AISSVLNDILSRLD | 77 | QYIKWPWYIWLGFI |
| 38 | AEIRASANLAATKM | 78 | VMVTIMLCCMTSCC |
| 39 | IRGWIFGTTLDSKTQSLL | 79 | LGKYEQYIKWPWYI |
| 40 | ITPGTNTSNQVAVL |  |  |
